# Left neck and right biceps muscle vibrations have similar effects on perceived body orientation

**DOI:** 10.1007/s00221-024-06994-6

**Published:** 2025-01-27

**Authors:** Britta Stammler, Hans-Otto Karnath

**Affiliations:** 1 Center of Neurology, Division of Neuropsychology, Hertie-Institute for Clinical Brain Research, University of Tübingen, Tübingen, Germany; 2 Center of Neurology, Department of Neurology and Stroke, Hertie-Institute for Clinical Brain Research, University Hospital Tübingen, Tübingen, Germany

**Keywords:** Spatial neglect, Hemineglect, Spatial attention, Proprioception, Muscle vibration, Subjective straight-ahead (SSA), Neglect rehabilitation

## Abstract

Vibrating muscles to manipulate proprioceptive input creates the sensation of an apparent change in body position. This study investigates whether vibrating the right biceps muscle has similar effects as vibrating the left posterior neck muscles. Based on previous observations, we hypothesized that both types of muscle vibration would shift the perception of healthy subjects’ subjective straight-ahead (SSA) orientation in the horizontal plane to the left. Such a finding would be extremely interesting for future treatment of spatial neglect, a disorder following right-sided stroke brain lesions. Twenty healthy participants (11 females, 9 males, aged 20–52) were tested under five conditions: baseline (no vibration), vibration of left neck muscles, vibration of right biceps with the arm fixed to the wall, vibration of right biceps with the arm lying on a table, and vibration of right triceps with the arm fixed to the wall. Participants had to align a laser pointer (by verbal instructions) with their perceived SSA position in complete darkness. ANOVA revealed significant SSA shifts with neck and biceps vibrations but not with triceps vibration. The largest leftward SSA shift occurred with right biceps vibration while the arm was lying on the table (-6.1°), followed by left neck muscle vibration (-6.0°), and right biceps vibration with the arm fixed to the wall (-5.4°). Post-hoc power analyses showed high power (> 0.98) for the significant differences compared to the baseline condition. The finding that right biceps vibration affects SSA perception similarly to left neck muscle vibration offers potential for clinical applications in treating spatial neglect. Future research should explore the therapeutic efficacy of vibrating the right biceps in neurological patients with spatial neglect.

## Introduction

Spatial attention is key to navigating space and interacting with objects and people. It relies on the complex integration of multisensory information, including visual, vestibular, and proprioceptive inputs, such as information from the retina, muscle spindles, and cupulae, which are integrated into higher-order, egocentric representations of space (Andersen 1997; Karnath 1994a, b, 1997). The dominant anatomical structure for this integration is the perisylvian network, consisting of the right superior/middle temporal, inferior parietal, insular and ventrolateral frontal cortex (Karnath and Dieterich 2006), interconnected by white matter fiber bundles.

Accordingly, experimental manipulation of sensory input to this system can induce kinaesthetic illusions. For example, vibrating muscles or their tendons to manipulate proprioceptive input activates muscle spindles, creating the sensation of limb movement (Eklund 1972; Goodwin et al. 1972a, b; Kito et al. 2006; McCloskey 1973; Roll and Vedel 1982; Schofield et al. 2015; Seizova-Cajic et al. 2007; White and Proske 2009) or apparent head movement by vibrating the neck muscles (review by Jamal et al. 2020), leading to apparent changes in body position. They occur due to the brain’s interpretation of altered proprioceptive signals from the body’s periphery. For example, if participants fixate on a stationary visual stimulus, vibratory manipulation of the posterior neck muscles can induce an illusory shift of the target to the opposite side of vibration (Biguer et al. 1988; Karnath 1994a, b, 1995; McIntyre and Seizova-Cajic 2007; Popov et al. 1999). The visual illusion is accompanied by a horizontal shift in the individual’s sense of own body orientation in relation to the visual surroundings, i.e. a shift of subjective straight-ahead (SSA) perception (Ceyte et al. 2006; Karnath et al. 1996, 2002; Leplaideur et al. 2016; Schindler and Kerkhoff 2004; Schindler et al., 2002). For example, with vibrating the neck muscles on the left side, the SSA shifts by an average of 4° to 10° toward the left in the horizontal plane (Biguer et al. 1988; Karnath et al. 1996, 2002; Schindler et al., 2002).

**Fig. 1:**
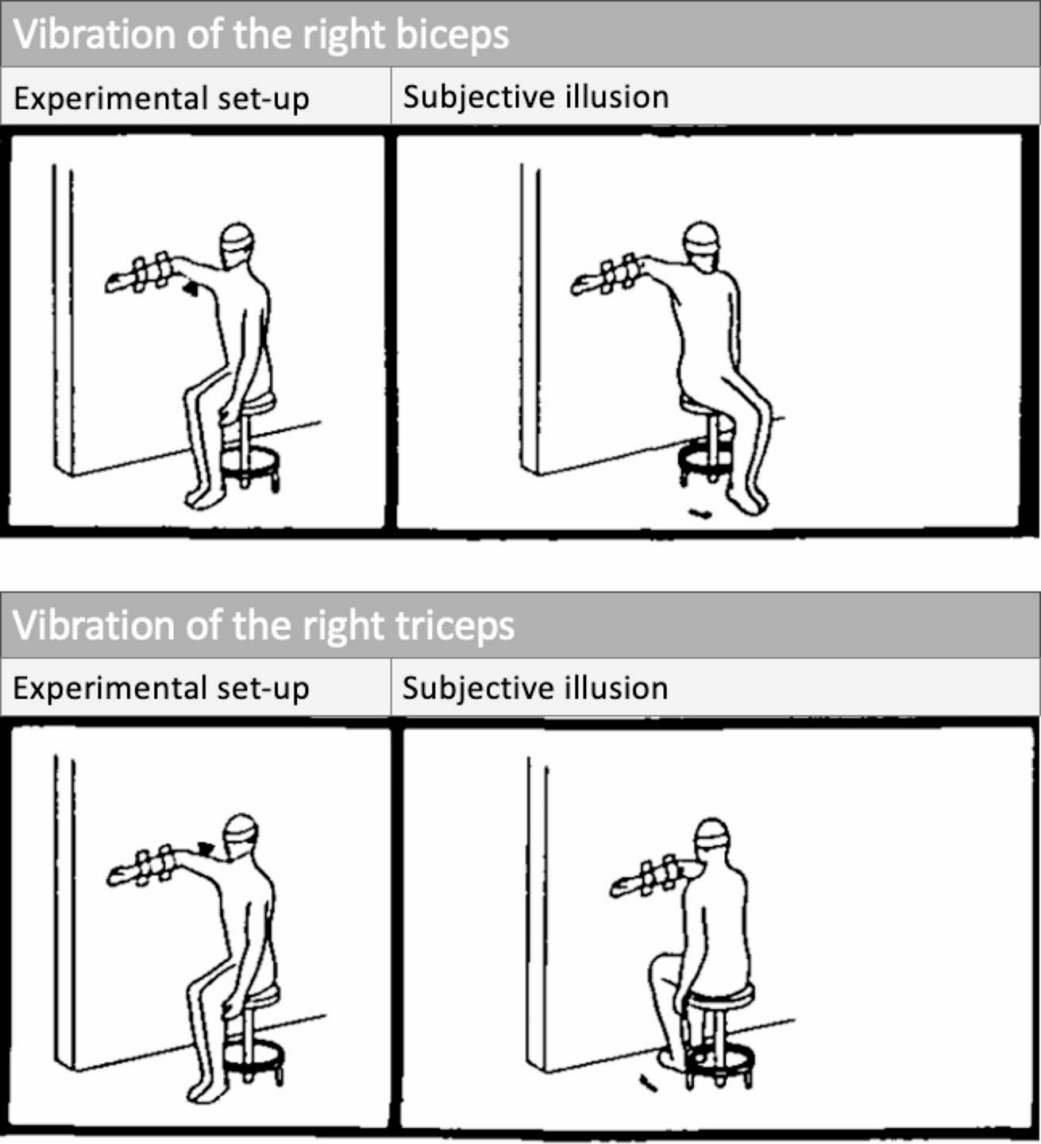
Lackner’s (1988) experimental design: The participant sat on a rotating chair, which was secretly locked in position, and the forearm was fixed to the wall. By vibrating the right biceps (top left picture), illustrated by a black triangle, participants felt the illusion that their body was moving to the left (top right picture). Vibrating the triceps (bottom left picture) provoked the opposite illusion, namely participants felt that their body was moving to the right (bottom right picture) (From Lackner 1988)

These observations are particularly intriguing, as it has become known that spatial neglect in neurological patients – i.e., stroke patients with an attentional bias towards the ipsilesional side and neglect of contralesional objects (Corbetta and Shulman 2011; Karnath and Rorden 2012) − is associated with a deviation in the SSA (Karnath 1994a, b). Furthermore, it turned out that neglect symptoms can be successfully treated by vibrating the left posterior neck muscles (Johannsen et al. 2003; Kamada et al. 2011; Karnath et al. 1993; Schindler et al., 2002). In this context, the early observations by Lackner (1988) are important. He conducted experiments on healthy subjects to highlight the role of proprioceptive inputs in altering the perception of body orientation. In one configuration, blindfolded participants sat on a rotating chair that was secretly locked in position by the experimenter. The right arm was fixed to the wall using an arm holder (Fig. 1). By vibrating the right biceps, 93% of the participants felt that their arm was stretching. However, as their forearm was fixed to the wall, they had the feeling that their whole body was turning to the left (by up to 90° counterclockwise) on the rotating chair. In fact, the physical orientation and configuration of the body did not change during vibration. Application of vibration to the triceps muscle led to an illusory perception of forearm flexion, having the opposite effect on subjects’ perception of body position; they now had the feeling that their body was turning against the wall (Fig. 1).

Based on these observations, the present study should investigate whether vibration of the right biceps has the same effect on the subjective perception of body orientation in the horizontal plane as it is known from vibration of left posterior neck muscles. Inspired by Lackner’s experiment, triceps vibration was included as a control condition. We asked whether left neck and right biceps muscle vibration affect the perception of healthy subjects’ SSA in the same manner, i.e. leads to a perceived shift of the body’s orientation in the horizontal plane by about 4° to 10° towards the left. An additional biceps vibration setup, with the arm resting on a table, was included because of its potential therapeutic relevance, as biceps vibration in this way would be practical and easy to perform in everyday clinical practice.

## Methods

### Participants

The study included 20 healthy participants, consisting of 11 females and 9 males, aged between 20 and 52 years (mean age = 25.4; SD = 6.8). None of the participants had a history of vestibular or oculomotor abnormalities; all participants were free from any neurological or psychiatric disorders. The study was approved by the ethics committee of Tübingen University and all participants provided their informed consent in accordance with the ethical standards of the 1964 Declaration of Helsinki.

### Apparatus and procedure

**Fig. 2:**
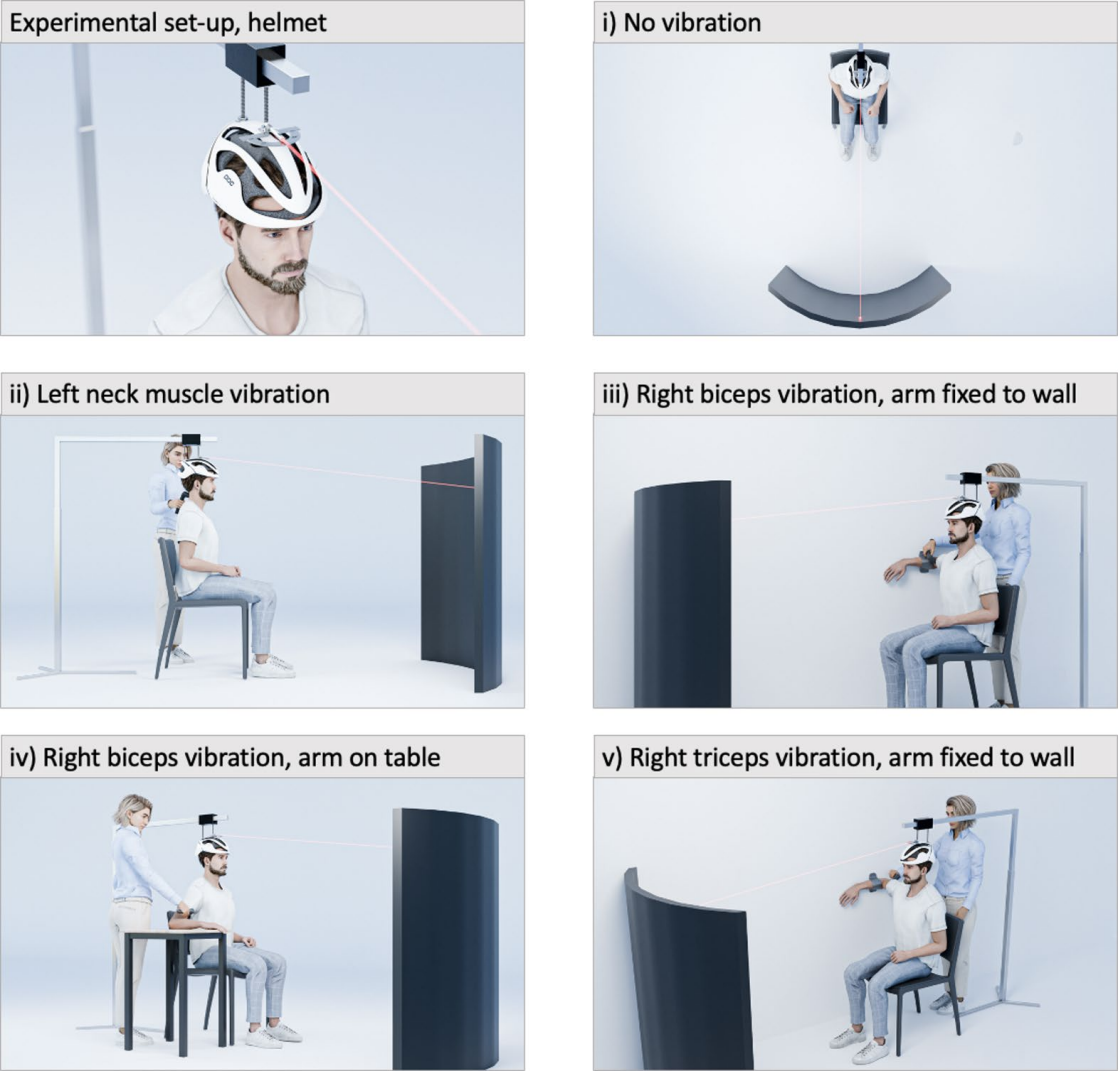
Experimental set-up with detailed illustration of the helmet with the laser pointer (top left) as well as the illustration of the five experimental conditions: **i**) without vibration (baseline), **ii**) with vibration of the left posterior neck muscles, **iii**) vibration of the right biceps while the arm was fixed to the wall, **iv**) vibration of the right biceps while the arm was lying on a table, and **v**) vibration of the right triceps while the arm was fixed to the wall

The experiment was conducted in complete darkness. The participants sat in an upright position on a chair, two meters in front of a semicircular panel (Fig. 2). They wore a helmet that was fixed to a static metal rod, ensuring that the participant could not move the head during the experiment. A laser pointer and a protractor were mounted on top of the helmet, projecting a red light spot onto the semicircular panel at the participant’s eye level. When the laser pointer was directed straight ahead, projecting a light spot directly in front of the participant, it corresponded to objective straight-ahead orientation (0°). Positive degrees of visual angle indicate a deviation to the right; negative degrees a deviation to the left.

For vibration, a device to be held in the hand (NOVAFON^®^, version: *Novafon Power* [https://novafon.com/]) was used with a frequency of 100 Hz and an amplitude of 3.8 mm. The head of the vibration device consisted of a flat disk with a diameter of 2.8 cm and was placed on the muscle belly. Participants were asked to determine their subjective straight-ahead (SSA) position in the following 5 experimental conditions (cf. Figure 2): (i) without vibration (baseline), (ii) with continuous vibration of the left posterior neck muscles, (iii) with continuous right biceps vibration while the arm was fixed to the wall, (iv) with continuous right biceps vibration while the arm was lying on a table, and (v) with continuous right triceps vibration while the arm was fixed to the wall. The experiment always began with the determination of the SSA in the baseline condition without vibration; the order of the following conditions ii) to v) was randomized. There was a pause of approximately one minute between conditions.

In each experimental condition, the SSA was determined four times per individuum. SSA measurement started with the red laser spot presented to one of 4 (pseudo)random positions: -15°, -10°, 10°, or 15°. The participant was asked to fixate on the light spot, and − through verbal feedback, such as “more to the right” or “more to the left” etc. − to adjust the light spot until it aligned with their subjective straight-ahead position. The experimenter was positioned behind the participant and physically manipulated the orientation of the laser according to the verbal instructions given by the participant (Fig. 2). The next trial of SSA measurement started with the red laser spot being presented to one of the remaining starting positions (-15°, -10°, 10°, or 15°). Results of the four SSA measurements were averaged.

Before the SSA measurement began, in each experimental condition it was verified whether or not the participant perceived horizontal visual motion of the laser spot when the vibration device was applied to the left posterior neck muscles, the right biceps (in the configuration where the arm was fixed to the wall), or the right triceps. The experimenter asked for the illusion in a directionally open, non-suggestive manner: “Do you notice any movement of the light spot?“.

### Statistical analysis

To evaluate the effects of vibration on motion illusion and subjective straight-ahead (SSA) perception, the following statistical tests were conducted for binary and continuous data using SPSS v.30 (IBMCorp., 2021): For the motion illusion, differences between the three vibration conditions (left neck muscles, right biceps, right triceps) were analyzed using a Cochran-Q-Test. Post-hoc pairwise comparisons were performed using McNemar tests with Bonferroni correction (α = 0.05/3 = 0.0167). For the subjective straight-ahead (SSA), a repeated-measures ANOVA with Bonferroni-corrected post-hoc comparisons (α = 0.05/10 = 0.005) was used to examine differences between the five conditions. Effect sizes were reported as partial eta squared (η²) for the ANOVA and Cohen’s d for pairwise comparisons.

## Results

### Motion illusion

With vibration of left posterior neck muscles, of right biceps, and of right triceps, the majority of participants experienced an illusion of movement of the stationary light spot. This number was highest with 90% for neck muscle vibration, followed by vibration of the biceps with 75% and of the triceps with 65%. The statistical evaluation of the potential influence of factor vibration conditions revealed a significant effect on the frequency of motion illusions (Cochran-Q(2) = 7.60, *p* = 0.022). Pairwise Bonferroni corrected post-hoc comparisons, however, did not reveal any significant differences (all *p* > 0.06).

### Subjective straight-ahead (SSA)

**Fig. 3:**
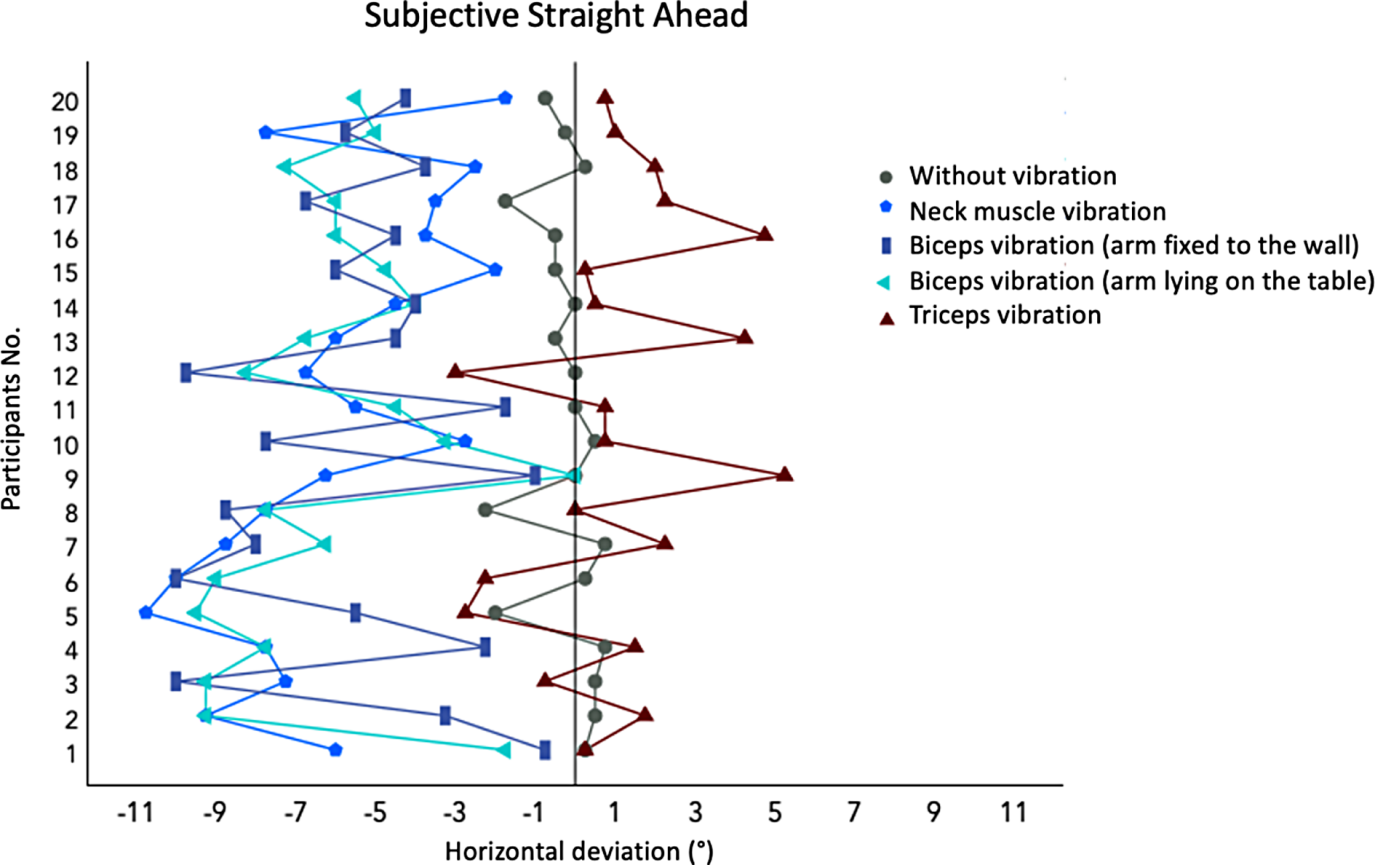
Individual SSA perception (averaged over 4 trials per subject) of the 20 participants in the baseline condition without vibration as well as conditions ii to v with vibration. The vertical black line shows the objective straight ahead orientation 0° in the horizontal plane

Figure 3 shows the individual SSA scores for all 20 participants for each of the five conditions; Table 1 shows the average scores and standard deviations for the whole group. In the baseline condition without vibration, a mean SSA close to 0 was observed (mean=-0.24, SD = 0.87). The largest shift of the SSA to the left was measured in the biceps vibration condition with the arm lying on the table, followed by the neck muscle vibration condition; in both these conditions we observed an average shift of approximately − 6° to the left.

The criteria for conducting an ANOVA for repeated-measures and factor experimental condition (no vibration, neck muscle vibration, biceps vibration with the arm fixed to the wall, biceps vibration with the arm lying on a table, triceps vibration with the arm fixed to the wall) were found to be met: The normal distribution was confirmed in all vibration conditions (Mauchly test: all *p* > 0.38); furthermore, the sphericity assumption was met (Shapiro-Wilk test: *p* = 0.74). The ANOVA revealed a significant result with a large effect size (F(4,76) = 61.61, *p* < 0.001, η^2^ = 0.76). Bonferroni corrected post-hoc comparisons (see Table 2) revealed significant differences in SSA position between the baseline condition without vibration and the condition with neck muscle vibration, as well as between the baseline condition and both conditions with biceps vibration (arm fixed to the wall, and arm lying on a table). For these significant differences, the post-hoc power analysis indicated a power exceeding 0.98, demonstrating sufficient sensitivity. No significant difference was found between the baseline condition and the condition with triceps vibration. However, in this case, the post-hoc power was relatively low (0.31).

**Table 1:** Descriptive data (mean [SD] and median [range]) for the SSA perception in the horizontal plane, averaged over the 20 participants in each of the five experimental conditions

Experimental condition	Horizontal deviation (°)	
	Mean(SD)	
No vibration (baseline)	-0.24 (0.87)	0.00 (-2.25 − +0.75)
Neck muscle vibration	-6.03 (2.70)	-6.13 (-10.75 − -1.75)
Biceps vibration (arm fixed to the wall)	-5.41 (2.93)	-5.00 (-10.00 − -0.75)
Biceps vibration (arm lying on the table)	-6.09 (2.56)	-6.13 (-9.50 − 0.00)
Triceps vibration	+ 0.98 (2.22)	+ 0.75 (-3.00 − +5.25)

**Table 2:** Post hoc comparisons for SSA in the five experimental conditions

		Mean difference	SE	t	Cohen’s d	p_bonf_
No Vibration	Neck muscle vibration	5.787	0.620	9.341	2.435	< 0.001
	Biceps vibration (Wall)	5.175	0.620	8.353	2.177	< 0.001
	Biceps vibration (Table)	5.85	0.620	9.442	2.461	< 0.001
	Triceps vibration	-1.212	0.620	-1.957	-0.51	0.54
Neck muscle vibration	Biceps vibration (Wall)	-0.612	0.620	-0.989	-0.258	1
	Biceps vibration (Table)	0.063	0.620	0.101	0.026	1
	Triceps vibration	-7	0.620	-11.299	-2.945	< 0.001
Biceps vibration (Wall)	Biceps vibration (Table)	-0.675	0.620	-1.089	-0.284	1
	Triceps vibration	-6.387	0.620	-10.31	-2.687	< 0.001
Biceps vibration (Table)	Triceps vibration	-7.062	0.620	-11.399	-2.971	< 0.001

*P*-value Bonferroni-corrected for comparing a family of 10

## Discussion

Participants most frequently perceived a visual illusion of light spot displacement during neck muscle vibration (90%), followed by biceps vibration (75%) and triceps vibration (65%). Our results are consistent with previous studies showing that, e.g., neck muscle vibration induces a visual illusion of light spot displacement in about 65–90% of cases (Biguer et al. 1988; Karnath 1994a, b, 1995; Leplaideur et al. 2016; Popov et al. 1999). Most importantly, we observed a significant deviation in the SSA perception with left neck muscle vibration (-6.03°) and both vibration conditions of the right biceps muscle (arm fixed to the wall: -5.41°; arm lying on a table: -6.09°). These shifts are in the range of previous observations of left neck muscle vibration, causing shifts between − 4° and − 10° (Biguer et al. 1988; Karnath et al. 1996, 2002; Schindler et al., 2002). This interesting, new finding indicates that right biceps vibration can influence the horizontal representation of body orientation in a comparable manner and direction as this has been observed for left neck muscle vibration.

The underlying mechanism of neck muscle vibration on SSA perception is explained by changes in the “head-on-trunk signal”. The information about muscle stretch (induced by vibration of the muscles), while head position remains physically unchanged, causes a perception of apparent leftward deviation of the trunk midline (around the earth-vertical head-body axis), thus shifting SSA perception (Biguer et al. 1988; Karnath et al. 1991). In parallel, the feeling of whole body turning to the left under vibration of the right biceps when the right arm is fixed to the wall (Lackner 1988) is explained by changes in the “limb-to-trunk signal”: the perceived extension movement of the fixated arm leads to the perceived rotation of the participants’ body (around the earth-vertical head-body axis) to the left. Our study shows that to alter SSA perception under this condition of “limb-to-trunk signal” manipulation, it is not necessary to fixate the right arm to the wall. To achieve this effect, it is essential for the participant to sit upright and keep the arm positioned laterally to the body while remaining physically still during the biceps vibration. The position of the arm, whether fixed laterally to a wall (cf. Lackner 1988) or lying flat on a table (cf. this study), does not appear to matter, as the mean horizontal deviations of SSA showed no significant difference in the present study.

Regarding right triceps vibration, we anticipated SSA to shift significantly to the right, compared to the baseline condition without vibration. This was observed numerically (see Table 1 above), but not statistically. One possible explanation for this finding could be differences in muscle spindle density and function between the biceps and triceps. The biceps brachii, involved in fine motor skills and precision tasks such as elbow flexion and forearm supination, indeed contains a higher density of muscle spindles compared to the triceps brachii (Banks 2006; Landin et al. 2017). The higher spindle density in the biceps might make this muscle more sensitive to proprioceptive manipulations, leading to a relatively stronger influence on SSA. Supporting this view, Lackner’s (1988) experiment demonstrated that triceps vibration resulted in fewer illusions compared to biceps vibration in three out of seven different body configurations. Notably, some of his subjects did not experience any illusion of flexion during triceps vibrations, while the illusion of extension was consistently reported during biceps vibrations (with the exception of one subject who reported no illusion in any of the configurations; this was always the same subject).

The present finding that right biceps vibration causes a shift in SSA (in a comparable manner as this has been observed for left neck muscle vibration) has important implications for clinical practice in neurological rehabilitation. Regarding the treatment of stroke patients with spatial neglect, vibration of the right biceps while the arm is lying (laterally of the body) on a table is easy to perform by therapists and could be even applied by patients themselves (e.g., after discharge from hospital). Simultaneous vibration of the right biceps and the left posterior neck muscles could also be an option to possibly enhance the therapeutic effect. Previous studies have shown that simultaneous stimulation of different proprioceptive and sensory input channels (such as neck muscle vibration, physical trunk rotation, and vestibular stimulation) involved in the creation of egocentric spatial coordinate systems can lead to additive effects, resulting in an increased shift in SSA and increased reduction of neglect symptoms (Karnath 1994a, b; Karnath et al. 1993, 1996; Wiart et al. 1997). Beyond vibration of left posterior neck muscles and right biceps, it might also be advantageous to include further muscles in such a combination therapy. For example, vibratory influence on trunk muscles might have additional impact on apparent position of the trunk. The obliquus externus abdominis muscle, e.g., plays a decisive role in trunk rotation. In trunk rotation to the left, the left abdominal muscle contracts and the right one stretches (Schünke, 2022). So it would be interesting to investigate whether vibration of the right obliquus externus abdominis muscle would also have an effect on SSA perception.

Beyond the (simultaneous) combination of vibrations from different muscles, it is also possible to optimize the vibration stimulus itself to enhance therapeutic effects. Vibration is characterized by its frequency and amplitude, and varying these parameters influences the activation level of muscle spindles in the vibrated muscles. This, in turn, affects the intensity of the kinesthetic illusion (Taylor et al. 2017). With respect to frequency, it turned out that a frequency between 80 and 100 Hz is optimal for activating muscle spindles and creating kinesthetic illusions (Eklund and Hagbarth 1966; Goodwin et al. 1972a, b; Kavounoudias et al. 2001; Roll et al. 1989; Taylor et al. 2017). In contrast, the effect of different amplitudes has received less attention in previous studies. Some studies did not specify the amplitudes used at all (Lackner 1988; Seizova-Cajic et al. 2007; White and Proske 2009). Other researchers reported using amplitudes of 0.2 mm (Roll and Vedel 1982), 1 mm (McCloskey 1973), 1.8 mm (Eklund 1972), or 6 mm (Kito et al. 2006). Schofield and colleagues (2015) conducted a study where they varied both the frequency and amplitude of the vibration and investigated the extent of the movement illusion following triceps and biceps vibration. They found that amplitude (ranging from 0.1 mm to 0.5 mm) had a more significant impact on the experience of the kinesthetic illusion compared to frequency; higher amplitudes resulted in a stronger effect. Consistent with this, Biguer (1988) reported that increasing the amplitude of neck muscle vibration led to greater deviations in SSA. Thus, the amplitude of 3.8 mm chosen in the present study appears to be large enough to visualize its effects on the displacement of the SSA by right biceps vibration, but it could possibly be optimized for therapeutic applications.

## Conclusion

We observed that right biceps vibration can influence the horizontal representation of body orientation in a comparable manner and direction as this has been observed for left neck muscle vibration. The next step would be to investigate whether right biceps vibration indeed has therapeutic benefits for neurological patients with spatial neglect, and to determine if these effects are long-lasting. Since left neck muscle vibration has already been proven effective and lasting in treating neglect symptoms (Johannsen et al. 2003; Kamada et al. 2011; Schindler et al., 2002), right biceps vibration could indeed offer a new therapy option that is easy to apply in both clinical and home settings.

## Data Availability

The data that support the findings of this study are available from the corresponding author upon reasonable request.
